# Frequency-Modulated Signal Measurement Using Closed-Loop Methodology

**DOI:** 10.3390/s22207822

**Published:** 2022-10-14

**Authors:** Xinglin Sun, Haojie Wu, Xinyue Tan, Wenrui Wang, Lingyun Ye, Kaichen Song

**Affiliations:** 1 College of Biomedical Engineering and Instrument Science, Zhejiang University, Hangzhou 310027, China; 2 School of Aeronautics and Astronautics, Zhejiang University, Hangzhou 310027, China

**Keywords:** frequency measurement, frequency-modulated signal, phase–frequency mapping, phase–frequency detector, negative feedback system

## Abstract

Frequency-modulated (FM) signals are widely used in sensing, measurement, and signal detection due to their strong anti-interference and easy transmission characteristics. Although the high-precision measurement methods for static signals are quite complete, the high-precision measurement methods for dynamic FM signals still need to be studied, and the measurement accuracy in the high-sampling system still has room for improvement. Traditionally, the equal-precision measurement method is widely applied in most scenarios. However, its accuracy is limited by the quantization error of ±1 word and the sampling gate time, making it difficult to improve the frequency measurement accuracy while ensuring a high sampling rate at the same time. In this paper, a high-precision feedback frequency measurement system with the capability to eliminate the quantization error of ±1 word is proposed. The proposed system consists of two stages, the rough measurement stage based on the equal-precision measurement method and the precise measurement stage based on the negative feedback tracking architecture using the phase–frequency detector (PFD) and direct digital synthesizer (DDS). The effectiveness and feasibility of the system are verified by both simulation and experiment. At the sampling rate of 2 kHz, the frequency measurement accuracy is improved by more than 30 dB.

## 1. Introduction

Frequency-modulated (FM) signals are widely used in the area of sensing, measurement, and signal detection due to their strong anti-interference ability, good anti-noise performance, and long transmission distance. In the design of high-performance sensors, the method of converting the physical quantity-to-frequency is mainly adopted [1,2]. Among them, the Optoelectronic Oscillator-based (OEO-based) optical fiber sensing system is widely studied. It converts the variation of physical quantities (such as length [3], strain [4,5], pressure [6], angular velocity [7], and temperature [8]) into the frequency variation of the microwave signal generated by OEOs. Ref. [9] maps the in-line position into the oscillating frequency shift and realizes precise positioning. In such optoelectronic sensing systems, the high-sensitivity and low-noise sensing principle contributes to high-precision detection, which can be realized by obtaining the physical quantity-to-frequency mapped information. It is obvious that the frequency measurement of the FM signal is the key stage that directly determines the detection performance.

Normally, the FM signal is conditioned at the front end, following which the traditional equal-precision frequency measurement method is applied to measure the tested signal [10]. In principle, the gate signal of the equal-precision measurement is synchronized with the tested signal to achieve the same detecting accuracy regardless of the frequency of the tested signal. During the gate time, the standard reference signal and the tested signal are counted at the same time, and then the frequency of the tested signal is obtained based on the mathematical relationship between the two count values. The detection accuracy of the traditional equal-precision measurement can be improved with a longer gate time and higher frequency of the standard reference signal. However, due to the high sensitivity of the sensor and high dynamically changing physical quantities, the instantaneous frequency of the FM signal changes rapidly and drastically. In this situation, the measuring rate must be increased to better obtain the waveform of the FM signal, which means an unavoidable decrease in the detecting accuracy. Thus, the relationship between the detecting accuracy and the measuring rate of the equal-precision measurement method is contradictory. In addition, the equal-precision frequency measurement method has the limitation of ±1 counting error [11]. In other words, using the equal-precision frequency measurement method solely cannot meet the requirements of high sampling and high precision at the same time. Therefore, there is an urgent need for a high-precision frequency measurement technology that can achieve wide dynamic range, high sampling rate, and high stability.

For the traditional frequency measurement technology, there are mainly two methods to eliminate the quantization error [12]. One method is to expand the quantization error and then perform second-stage quantization, such as analog interpolation [13], delay line interpolation, etc. These methods have been verified by experiments and can improve the accuracy of frequency measurement. In [14,15], Polish scholars used the TDC method in FPGA to realize the measurement of frequencies up to 200 MHz, and the maximum frequency measurement error within 1 s is 2.4 Hz. In [16], Y.H Ge et al. used the TDC-GP22 chip to achieve an extended measurement of ±1 counting error. Under the condition of a sampling rate of 10 Hz, the frequency measurement resolution is smaller than 0.01 Hz. In [17], the National Timing Center of the Chinese Academy of Sciences developed a time–frequency measurement device based on an FPGA chip and a TDC-GP21 chip, in which case the measurement resolution can reach 13 ps. However, since the above-mentioned methods still adopt electronic counting methods to measure the frequency, the quantization error still exists despite the second-stage quantization.

Another method is to convert the counting error into other physical quantities, such as amplitude and phase. Then, based on the relationship between these physical quantities and frequency, the frequency measurement is achieved indirectly. This kind of method fundamentally eliminates the quantization error. In [18], the Finnish scholar proposed a frequency measurement method based on time-amplitude conversion, referred to as TVC (Time-to-Voltage Converter) method in 1991, which eliminated the quantization error in principle. Additionally, in the 1990s, the American Stanford company developed the SR620 frequency meter by using the TVC method, which achieved a very high resolution (25 ps) and a measurement range of up to 1.3 GHz. In addition, scholars proposed a method to improve the frequency measurement accuracy by using the group phase difference. In [19], Zhou Wei et al. studied the law of phase difference between different frequency signals in the 1990s, and proposed the phase detection based broadband frequency measurement technology for the first time. However, the phase coincidence point of this method is difficult to capture, and the dynamic performance is poor, making it unsuitable for the FM signal measurement of OEO output. Therefore, how to fundamentally solve the ±1 counting error and realize the high-precision frequency measurement technology with high sampling rate, wide frequency band, and high stability has become a major problem in the field of electronic measurement technology.

Aiming at the problem of the ±1 counting error existing in traditional frequency measurement technology at a high sampling rate, this paper proposes a high-precision feedback frequency measurement system based on PFD and DDS by analyzing the relationship between the frequency difference and phase difference. This method eliminates the quantization error commonly existing in frequency measurement in principle and ensures measurement accuracy even at a high sampling rate.

The high-precision feedback frequency measurement system is explained and detailed in Section 2. The simulation results of the proposed system are presented in Section 3. Experiments and measured results are demonstrated in Section 4. Conclusions are summarized in Section 5.

## 2. High-Precision Frequency Measurement System Based on Negative Feedback Control Structure

### 2.1. Overall Structure of the High-Precision Frequency Measurement System

**Figure 1:**
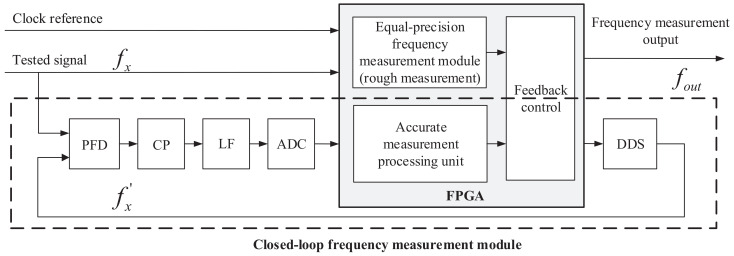
Overall structure diagram of the high-precision frequency measurement system based on negative feedback control.

The high-precision frequency measurement system proposed in this paper is mainly composed of two modules: the equal-precision frequency measurement module and the accurate closed-loop frequency measurement module. The block diagram of the overall structure design of the system is shown in Figure 1. The equal-precision measurement module is realized in FPGA based on the traditional equal-precision measurement method. It performs the rough measurement on the tested signal ($f_{x}$), and controls the DDS to generate an initial reference signal ($f_{x}^{\prime }$) according to the rough measurement result. The accurate closed-loop frequency measurement module is a negative feedback structure composed of a phase–frequency detector (PFD), a charge pump (CP), a loop filter (LF), analog-to-digital converter (ADC), field programmable gate array (FPGA), and direct digital synthesizer (DDS).

The two key components in the entire frequency measurement system are the PFD and DDS. The PFD is responsible for the precise phase measurement of the tested signal ($f_{x}$), working as the detection unit. The DDS generates a reference signal ($f_{x}^{\prime }$) that can precisely track the tested signal ($f_{x}$) according to the detection output of the PFD, working as the actuator unit. Owing to the two components, the system takes advantage of the fact that the PFD can realize high phase-to-voltage gain, which means it is more sensitive to small phase differences. Additionally, the system features the advantages of the negative feedback system based on DDS, which can achieve high control precision and good system stability. On the basis of the phase-to-voltage transformation method, the quantization error of the system is eliminated in principle, ensuring the detecting accuracy of frequency measurement in a high-sampling scenario. In addition, the wide dynamic range of the frequency measurement is realized by the DDS.

The basic working principle of the proposed frequency measurement system above is to achieve fast frequency tracking of the tested signal and realize high-sampling high-precision frequency at the same time.

Firstly, the equal-precision frequency measurement method is used to roughly measure the frequency of tested signal $f_{x}$.

Secondly, DDS automatically synthesizes the frequency of $f_{x}^{\prime }$ according to the measured result obtained by the rough measurement part.

Thirdly, $f_{x}$, $f_{x}^{\prime }$ are sent to the PFD for frequency and phase detection. Together with the CP, the phase and frequency error between $f_{x}$ and $f_{x}^{\prime }$ is transformed into the current information, which is proportional to the phase and frequency error. Then, through the active LF, the current-to-voltage stage is accomplished, and the voltage signal is sampled by ADC.

Finally, the FPGA processes the digital data acquired by ADC to obtain the precise phase and frequency error between $f_{x}$ and $f_{x}^{\prime }$ and transmit the configuration information to the DDS. The DDS dynamically adjusts its output $f_{x}^{\prime }$ according to the configuration information, realizing the closed-loop feedback control. The high-precision frequency measurement result is directly obtained by reading the DDS configuration information since $f_{x}^{\prime }$ is tracking and locked on $f_{x}$.

### 2.2. Theoretical Analysis of Phase–Frequency Conversion Measurement Method

The accurate frequency measurement module uses the relationship of the phase–frequency conversion between two signals to achieve a precise frequency measurement. The analysis of the phase–frequency conversion relationship of the signal is presented in this section.

**Figure 2:**
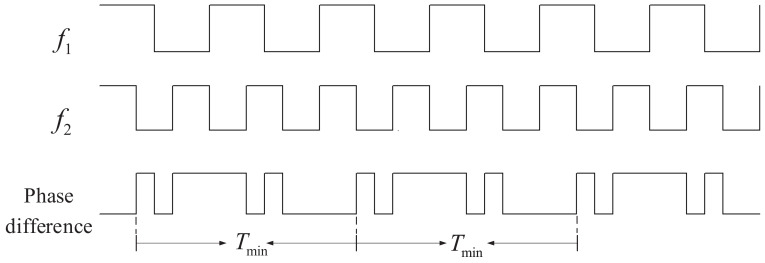
Variation of phase difference between two signals with a fixed-frequency difference.

Given two signals $f_{1}$ and $f_{2}$ with a fixed-frequency difference, if they can be expressed as $f_{1}=Af_{max}$ and $f_{2}=Bf_{max}$, where A≠B and A, B are two mutually prime positive integers, then $f_{max}$ is considered to be their greatest common factor frequency. $T_{min}$ is defined as the least common multiple period ($T_{min}=1/f_{max}$). It can be seen from Figure 2 that, ideally, the phase difference variation law of two signals with a fixed-frequency difference varies with $T_{min}$ as the period [20], as expressed in (1) and (2). At the start and end point of $T_{min}$, the phase of signals $f_{1}$ and $f_{2}$ are the same, which is called the phase coincidence point. It can be noticed that within the $T_{min}$ period, the phase difference does not change regularly or continuously, that is, the change in the phase difference is disordered. In other words, the relationship between the phase difference and the frequency is complex and not applicable to real-time measurement.

(1) $$T_{min}=\frac{1}{f_{1}/A}=AT_{1}$$

(2) $$T_{min}=\frac{1}{f_{2}/B}=BT_{2}$$

**Figure 3:**
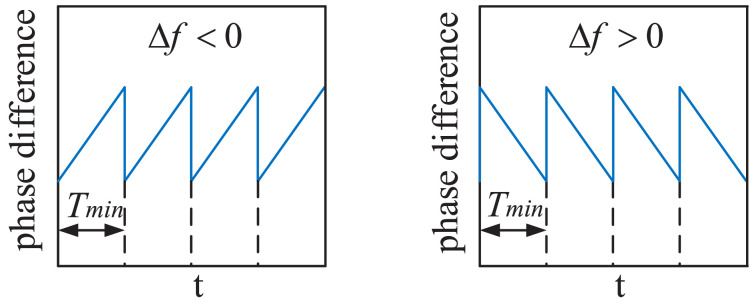
Variation law of phase difference between two signals with a small fixed-frequency difference.

However, when the frequency difference Δf of the two signals is very small (Δf→0, $f_{1}\approx f_{2}$) and Δf≠0, the variation law of the phase difference between the two signals with a fixed-frequency difference is ordered and linear. It can be seen in Figure 3 that within the $T_{min}$ period when the frequency difference Δf of the two signals is very small, it can be approximated that their phase differences are proportional to the frequency difference and the time interval. According to the definition of frequency and phase, within the time interval of Δt, the phases of $f_{1}$ and $f_{2}$ can be expressed as follows:

(3) $$\varphi_{1}=\int_{0}^{\Delta t}f_{1}dt=f_{1}\cdot \Delta t$$

(4) $$\varphi_{2}=\int_{0}^{\Delta t}f_{2}dt=f_{2}\cdot \Delta t$$

Combining Equations (3) and (4), the phase difference between $f_{1}$ and $f_{2}$ can be expressed as:

(5) $$\Delta \varphi =f_{1}\cdot \Delta t-f_{2}\cdot \Delta t=\int_{0}^{\Delta t}\Delta fdt=\Delta f\cdot \Delta t$$

**Figure 4:**
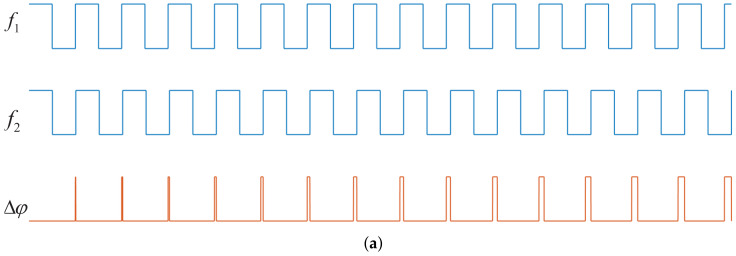

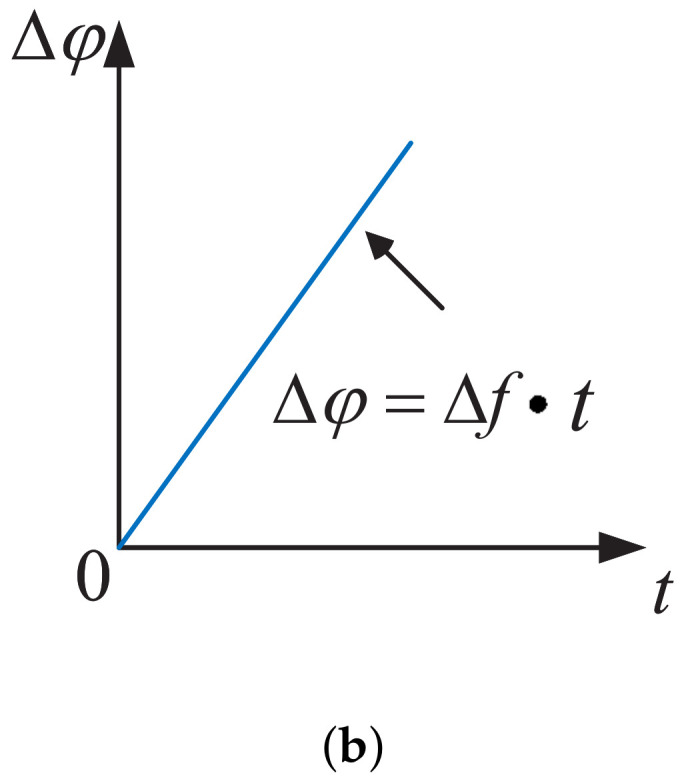
Variation of phase difference between two signals with a small fixed-frequency difference: (**a**) Schematic diagram of the relationship between the phase difference and time. (**b**) Schematic diagram of phase–frequency mapping.

To sum up, considering two signals $f_{1}$ and $f_{2}$ with a fixed-frequency difference, when the frequency difference Δf of the two signals is very small (Δf→0, $f_{1}\approx f_{2}$) and Δf≠0, the phase difference variation law is shown in Figure 4a. During the time interval of $T_{min}$, the phase difference of the two fixed-frequency difference signals changes with time, taking Δf as the slope coefficient, as shown in Figure 4b. Therefore, the high-precision frequency measurement can be transformed into the high-precision measurement of phase difference between the tested signal ($f_{x}$) and the reference signal ($f_{x}^{\prime }$), when the frequency of $f_{x}$ is close to $f_{x}^{\prime }$. However, in most scenarios, the frequency of the reference signal is fixed and it is impossible to meet this requirement. Thus, the detecting range will be greatly limited. To address this issue and apply the phase–frequency conversion measurement method, a dynamically adjustable reference signal is necessary. Its detailed description is presented in Section 2.3.2 below.

### 2.3. Theoretical Analysis of Accurate Frequency Measurement Module

#### 2.3.1. High-Precision Phase Detection

**Figure 5:**
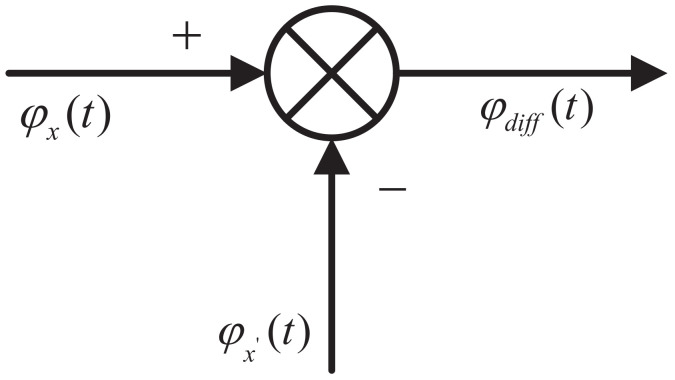
Mathematical model of the phase–frequency detector (PFD).

As the core component of the accurate frequency measurement module, the PFD is used to detect the phase difference between the input signal and the reference signal, and then convert the phase difference into a voltage signal. The mathematical model of the PFD is illustrated in Figure 5.

The charge pump (CP) in the PFD converts the phase detector’s digital outputs to appropriate current signal ($I_{CP}$), whose magnitude is proportional to the phase difference between the two input signals, and the direction is related to the sign of the phase difference. The relationship between the output current can be expressed in (6). $I_{Full}$ is the given maximum CP gain current, which defines the gain of PFD in amps/radians.

(6) $$I_{CP}=\frac{\Delta \varphi }{4\pi }I_{Full}$$

**Figure 6:**
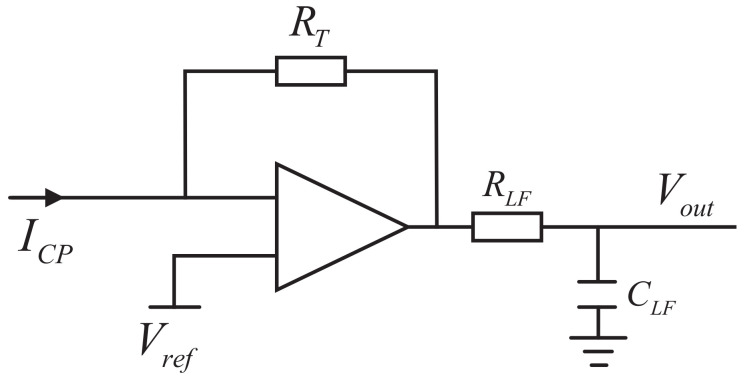
Schematic diagram of the current-to-voltage transformation.

Therefore, a widely used trans-impedance amplifier (TIA) can be applied after the output of the PFD. The schematic diagram of the current-to-voltage transformation is shown in Figure 6.

The relationship between the input current ($I_{CP}$) and the output voltage ($V_{out}$) in the S-domain can be expressed in (7).

(7) $$\begin{array}{cc} V_{out}\left(s\right) & =I_{CP}\left(s\right)\cdot R_{T}\cdot F\left(s\right)=I_{CP}\left(s\right)\cdot R_{T}\cdot \frac{1}{sR_{LF}C_{LF}+1}\\ \; & =\frac{I_{Full}R_{T}\Delta \varphi }{4\pi }\cdot \frac{1}{sR_{LF}C_{LF}+1} \end{array}$$

where
F(s)is the transfer function of the loop filter;$R_{LF}$, $C_{LF}$constructs the one-order RC low-pass filter;$R_{T}$is the feedback resistor in the TIA stage.

Combining (5)–(7), the average frequency difference between the two signals ($f_{x}$ and $f_{x}^{\prime }$) within the period of Δt is expressed in (8):

(8) $$\Delta f=\frac{\Delta \varphi }{\Delta t}=\frac{4\pi V_{out}}{I_{Full}R_{T}\Delta t}$$

It can be known from (8) that if the time interval Δt and the voltage output at the TIA $V_{out}$ are known, the Δf can be obtained. Since the reference signal $f_{x}^{\prime }$ is known, the frequency of the tested signal ($f_{x}$) can be obtained as:

(9) $$f_{x}=f_{x}^{\prime }+\Delta f=f_{x}^{\prime }+\frac{4\pi V_{out}}{I_{Full}R_{T}\Delta t}$$

#### 2.3.2. Negative Feedback Control System

In the accurate frequency measurement module, a negative feedback control system is proposed. The frequency difference between the two input signals detected at the PFD is used as the control information to dynamically configure the DDS. The DDS automatically adjusts the frequency of its output signal, decreasing the frequency difference between the reference signal $f_{x}^{\prime }$ generated by DDS and the tested signal $f_{x}$. Therefore, an accurate frequency tracking system is formed. Generally, two problems can be solved based on the negative feedback control system, compared with the open-loop measurement.

**Figure 7:**
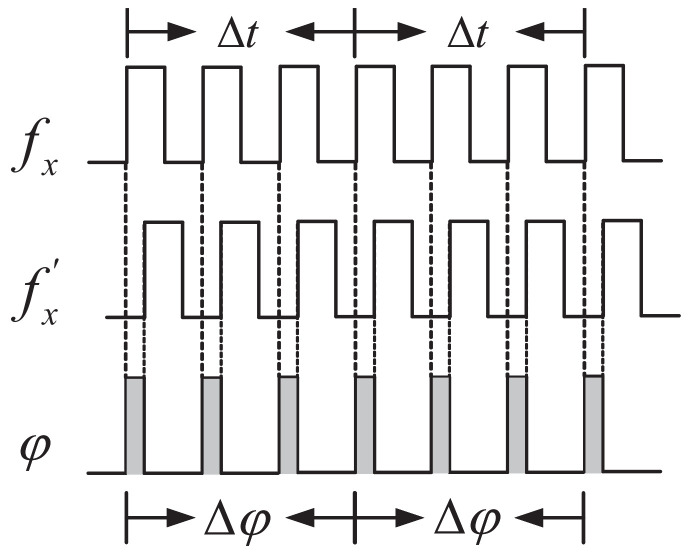
Variation law of phase difference between two signals with equal frequency and different initial phase.

(1) During the measurement procedure, there exists certain randomness in the initial phase of the reference frequency signal $f_{x}^{\prime }$ generated by the DDS and the tested signal $f_{x}$. If the open-loop measurement is performed directly, even if the frequencies of $f_{x}^{\prime }$ and $f_{x}$ are the same, there still exists a difference between the initial phase, as shown in Figure 7. In this situation, supposing the initial phase difference between the two signals is $\varphi_{0}$, the frequency result is expressed in (10). There will be an error of $\varphi_{0}/\Delta t$ generated in the measurement of the frequency, failing to achieve the purpose of precise frequency measurement.

(10) $$f_{x}=f_{x}^{\prime }+\Delta f=f_{x}^{\prime }+\frac{\varphi_{0}}{\Delta t}\neq f_{x}^{\prime }$$

(2) During the open-loop measurement, the input test signal $f_{x}$ will be an FM signal with a certain dynamic range, that is, the frequency of $f_{x}$ will have a frequency fluctuation compared with a reference signal with a fixed frequency. When $f_{x}$ changes drastically, there could be a big frequency difference between $f_{x}$ and $f_{x}^{\prime }$. The phase–frequency conversion measurement method described in Section 2.2 is no longer applicable since the relationship between the phase difference and frequency is disordered. What is more, limited by the phase detection range of the PFD, even a small frequency difference will generate a big phase difference as the time interval increases. When the accumulated phase difference during a sample period exceeds several cycles, without the information of exceeded cycles, the phase detection is inaccurate, and the measuring performance of the entire system is badly degraded.

Therefore, the negative feedback control system proposed in this paper tracks the steady-state phase difference between $f_{x}$ and $f_{x}^{\prime }$ to 0. That is to say, under the ideal situation, the entire accurate frequency measurement module can track the change in the tested signal $f_{x}$ accurately, eliminating the counting error of ±1 word in principle and ensuring the detecting dynamic range.

### 2.4. Analysis of the System Theoretical Frequency Measurement Accuracy

**Figure 8:**
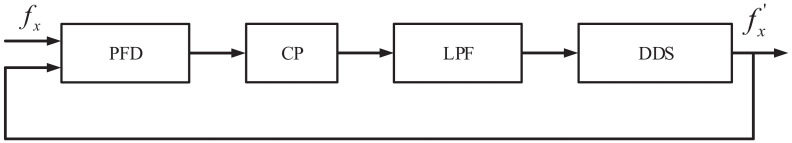
The structure of the accurate frequency measurement module using closed-loop feedback control.

The structure of the accurate frequency measurement module using closed-loop feedback control is shown in Figure 8. The theoretical frequency measurement accuracy of this structure depends on the phase tracking accuracy between the reference signal $f_{x}^{\prime }$ and the tested signal $f_{x}$. The mathematical model of the PFD, CP, and LF is described in Section 2.3.1.

Analogous to the voltage-controlled oscillator (VCO) in a phase-locked loop, DDS plays the same role in this closed-loop system. DDS adjusts the frequency of its output signal according to the phase difference measured by the PFD. The reference signal $f_{x}^{\prime }$ generated by DDS can be expressed in (11).

(11) $$f_{x}^{\prime }\left(t\right)=f_{0}+K_{o}u_{c}\left(t\right)$$

where
$f_{0}$is the initial reference signal;$u_{c}$is the voltage representing the phase difference;$K_{o}$is the gain coefficient.

The output of DDS is fed back to the PFD to detect the phase difference between $f_{x}^{\prime }$ and $f_{x}$, which means there exists a conversion between phase and frequency. So, the mathematical model of DDS has an integration stage to transform the frequency into phase, corresponding to an integration factor 1/s in the S-domain. Then, the transfer function of DDS is expressed in (12).

(12) $$\theta_{o}\left(s\right)=\frac{K_{o}}{s}$$

**Figure 9:**
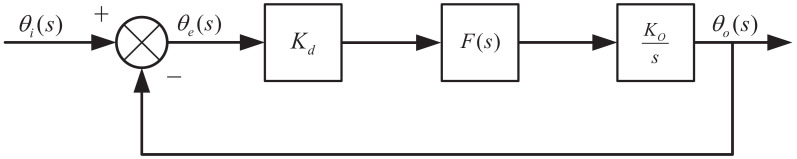
The simplified mathematical structure of the phase-transfer model.

Transform all the components in Figure 8 into mathematical models, the phase-transfer model of the whole loop can be simplified and shown in Figure 9. Similar to the structure of the phase-locked loop, the open-loop transfer function of the accurate frequency measurement module can be expressed in (13):

(13) $$H_{o}\left(s\right)=K_{d}F\left(s\right)K_{o}/s=\frac{I_{Full}R_{T}K_{o}F\left(s\right)}{4\pi s}$$

where
$K_{d}$is the phase gain coefficient of the PFD;F(s)is the transfer function of the loop filter;$H_{o}\left(s\right)$is the open-loop transfer function.

The closed-loop transfer function of the system can be expressed in (14):

(14) $$\begin{array}{cc} H(s) & =\frac{\theta_{o}\left(s\right)}{\theta_{i}\left(s\right)}=\frac{H_{o}\left(s\right)}{1+H_{o}\left(s\right)}=\frac{K_{d}K_{o}F\left(s\right)}{s+K_{d}K_{o}F\left(s\right)} \end{array}$$

Assuming *K* is the loop gain, then:

(15) $$K=K_{d}K_{o}$$

(16) $$\begin{array}{cc} H(s) & =\frac{\theta_{o}\left(s\right)}{\theta_{i}\left(s\right)}=\frac{H_{o}\left(s\right)}{1+H_{o}\left(s\right)}=\frac{KF(s)}{s+KF(s)} \end{array}$$

The transfer function of the phase error $\theta_{e}\left(s\right)$ in the loop can be obtained and expressed in (17):

(17) $$H_{e}\left(s\right)=\frac{\theta_{e}\left(s\right)}{\theta_{i}\left(s\right)}=\frac{1}{1+H_{o}\left(s\right)}=\frac{s}{s+KF(s)}$$

To obtain the steady-state phase error of the frequency measurement system, the inverse Laplace Transform is applied to obtain the following equations.

(18) $$\theta_{e}\left(s\right)=\theta_{i}\left(s\right)H_{e}\left(s\right)$$

(19) $$\theta_{e}\left(\infty \right)=\lim_{t\to \infty }\theta_{e}\left(t\right)=\lim_{s\to 0}s\theta_{i}\left(s\right)\frac{s}{s+KF(s)}$$

From (19), we can see that the steady-state tracking phase error of the system is related to the loop filter F(s) and the input signal $\theta_{i}\left(s\right)$. A one-order low-pass filter is used in the paper. Its transfer function can be expressed as:

(20) $$F\left(s\right)=\frac{1}{1+sRC}$$

The input test signal can be regarded as a phase step signal and expressed in (21):

(21) $$\theta_{i}\left(s\right)=\frac{1}{s}$$

Combining (19)–(21), the steady-state error transfer function of the accurate frequency measurement module can be expressed as (22):

(22) $$\theta_{e}\left(\infty \right)=\lim_{t\to \infty }\theta_{e}\left(t\right)=\lim_{s\to 0}s\theta_{i}\left(s\right)\frac{s}{s+KF(s)}=\frac{s}{s+\frac{K}{1+SRC}}=0$$

Therefore, the steady-state phase error between the tested signal and the tracking reference signal can reach zero. That is to say, under the ideal situation, the entire accurate frequency measurement module can dynamically adjust the reference signal $f_{x}^{\prime }$ to track the change in the tested signal accurately without error. Then, the frequency of $f_{x}$ can be directly obtained from the known reference frequency $f_{x}^{\prime }$ generated by the DDS, which is expressed in (23).

(23) $$f_{x}\left(t\right)=f_{x}^{\prime }\left(t\right)=\frac{FTW(t)}{2^{N}}f_{clk}$$

where
FTW is the frequency tuning word used to configure the output of DDS;*N*is the number of bits in the phase accumulator;$f_{clk}$is the system clock running in DDS.

The accuracy of the frequency measurement result depends on the accuracy of the reference signal generated by the DDS. Without considering the influence of output spurs, the theoretical output accuracy of DDS depends on the noise performance of the system clock and the number of bits in the phase accumulator. Since the frequency tuning word FTW is taken as an integer in this paper, ideally, the frequency resolution of the accurate frequency measurement module is expressed in (24):

(24) $$e_{precise}=\frac{\left|\Delta FTW\right|}{2^{N}}f_{clk}\geq \frac{f_{clk}}{2^{N}}$$

## 3. Simulation

In order to verify the effectiveness of the frequency measurement method proposed in this paper, a theoretical simulation model of the entire system is built in Simulink. As shown in Figure 1, the high-precision frequency measurement system based on a negative feedback control structure combines the rough measurement result from the equal-precision frequency measurement module and the precise measurement result from the accurate closed-loop frequency measurement module.

The equal-precision measurement method is adopted to achieve a rough measurement of the tested signal $f_{x}$, and then the accurate frequency measurement module uses the PFD to achieve high-precision frequency measurement. Since the PFD has a certain range of phase detection (−2π,2π), the system needs to quickly track the tested signal when performing the accurate frequency measurement to prevent the PFD from entering the saturation region, thereby ensuring the accuracy of frequency measurement. The control part of the accurate frequency measurement module adopts the classic proportional–integral–derivative (PID) control. In addition, during the first rough measurement, the accurate frequency measurement module should be disabled since the initial frequency output of the DDS is not obtained. Then, the accurate frequency measurement module is enabled after the first rough measurement is accomplished.

**Figure 10:**
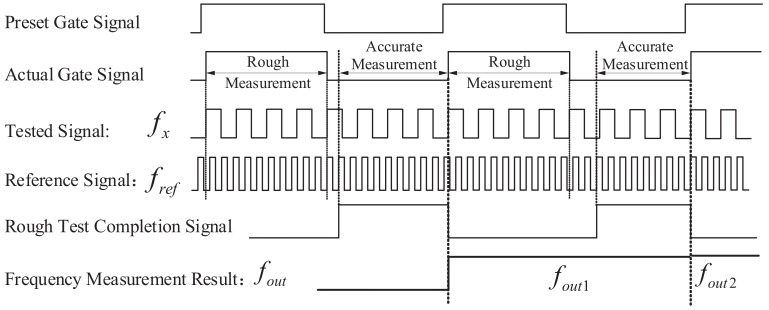
Control timing diagram of the proposed high-precision frequency measurement system based on negative feedback control structure.

The timing diagram of the entire frequency measurement system is shown in Figure 10. The preset gate signal, actual gate signal, and the reference signal $f_{ref}$ are used to realize the equal-precision frequency measurement, providing a rough measurement result of $f_{x}$. After the rough test completion signal is pulled high, the accurate measurement module is enabled, following the rough measurement.

Due to the limitation of the computer’s computing power, the simulation model is simplified. The related parameters are listed in Table 1.

When the gate frequency of the equal-accuracy frequency measurement module is 1 Hz, and the reference frequency is 200 kHz, the frequency accuracy of the equal-precision measurement method is theoretically calculated as [21]:

(25) $$\delta_{rough}=\frac{\left|\Delta N_{ref}\right|}{N_{ref}}\geq \frac{1}{N_{ref}}=\frac{1}{T_{Gate}\cdot f_{ref}}=10^{-5}$$

(26) $$e_{rough}=\delta_{rough}\cdot f_{x}=0.1\;\mathrm{Hz}$$

That is, the frequency measurement result of the tested signal using the equal-precision measurement method is 10 kHz ± 0.1 Hz. In comparison, theoretically, the frequency measurement accuracy of the accurate measurement module is determined by the resolution of the DDS. Under the given simulation conditions, the frequency accuracy can be expressed as:

(27) $$e_{precise}=\frac{64,000\;\mathrm{Hz}}{2^{26}}\approx 0.001\;\mathrm{Hz}$$

It can be seen from (25)–(27) that theoretically, the relative error of equal-precision measurement depends on the gate time $T_{Gate}$ and the frequency of the reference signal $f_{ref}$. When the sampling rate rises, the gate time will decrease, leading to degraded measurement accuracy. The only way to solve this issue is to increase the frequency of the reference signal, which cannot be increased without limit. Instead, the ideal measurement accuracy is independent of the sampling time, making it keep a high-precision detection even in a high sampling scenario.

**Figure 11:**
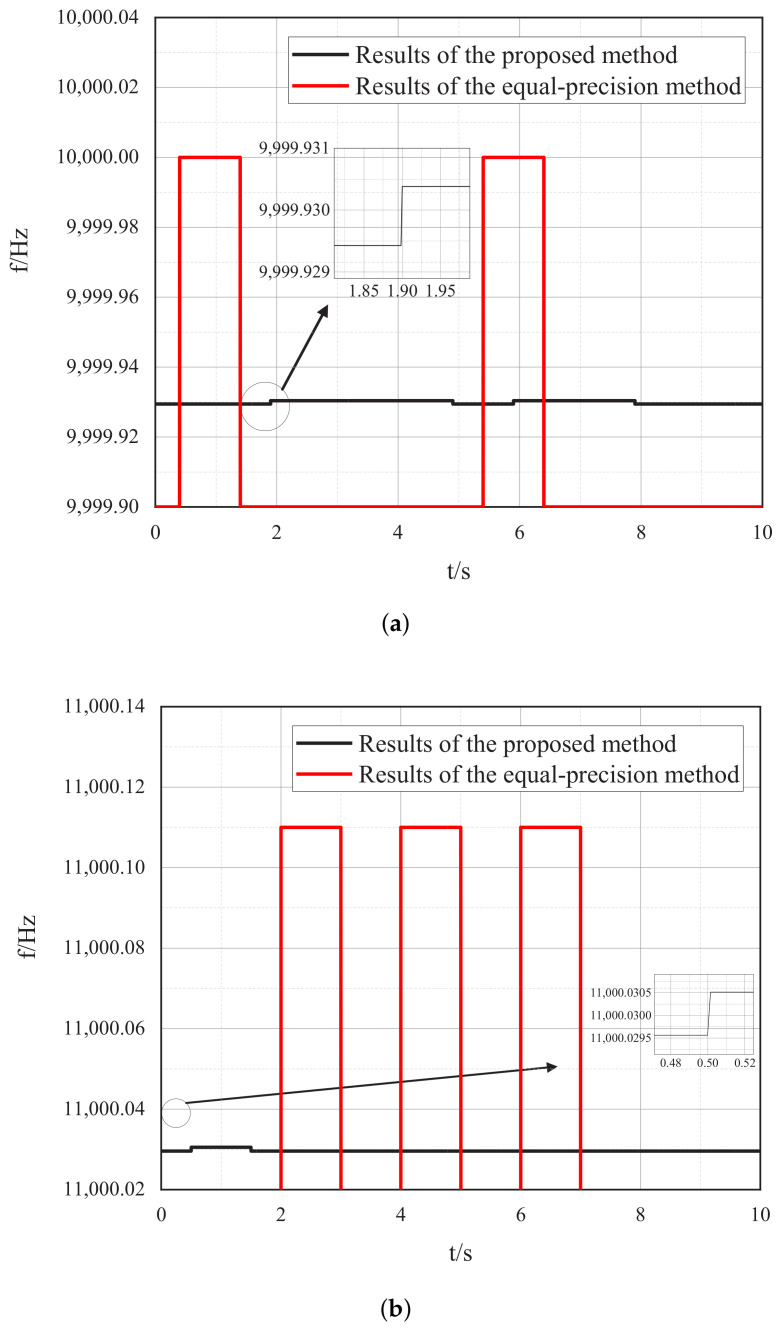
Simulation results of the proposed method and the equal-precision measurement method: (**a**) Tested signal: $f_{x}$ = 9999.930 Hz. (**b**) Tested signal: $f_{x}$ = 11,000.030 Hz.

Under the same sampling time of 1 s, the two groups of signals with the frequency of 9999.93 Hz and 11,000.030 Hz are measured using the proposed method and the equal-precision method, respectively. The simulated frequency measurement results are shown in Figure 11a,b.

The comparison table, shown in Table 2, lists the frequency measurement errors of the high-precision frequency measurement system proposed in this paper and the equal-precision frequency measurement method.

Through the comparison of the above table, it can be found that under the same gate time, the frequency accuracy of the high-precision frequency measurement system proposed in this paper is higher than that of the traditional equal-precision frequency measurement method. The relative error of the proposed method is less than $1\times 10^{-7}$ with the gate time of 1 s, while the relative error of the equal-precision measurement method with the same gate time is 1 × 10${}^{-5}$. The frequency measurement accuracy of the proposed method is about 30 dB better than that of the equal-precision method under the given simulation conditions.

## 4. Experiment and Validation

**Figure 12:**
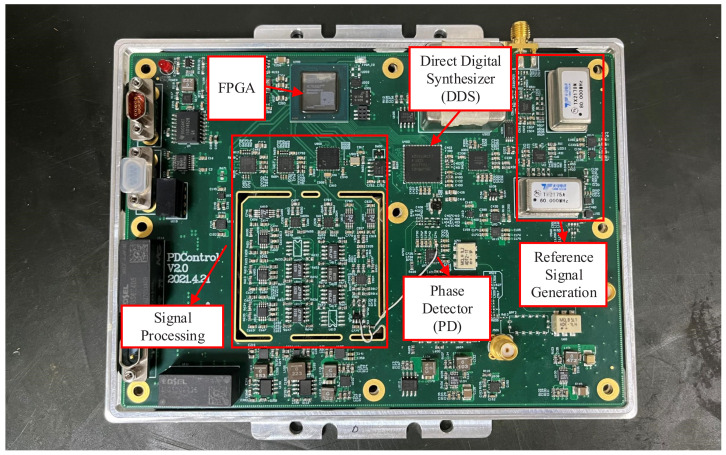
Photograph of the experimental board.

**Figure 13:**
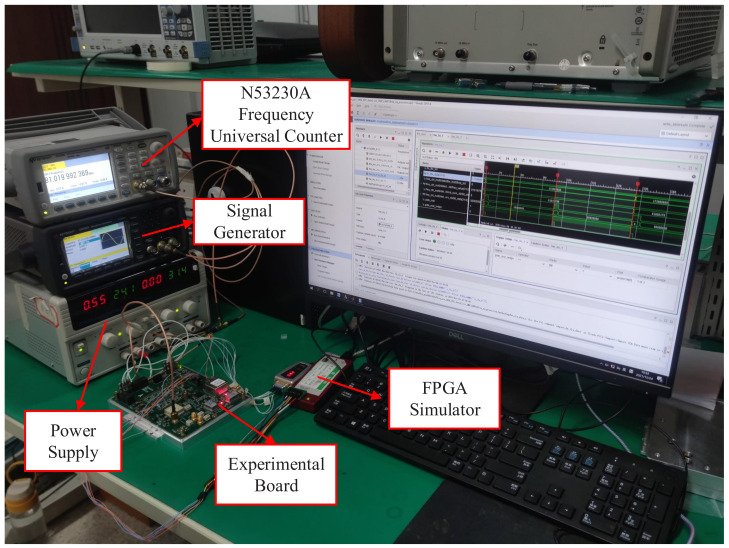
Photograph of conducting testing experiments.

The experimental board and test environment are shown in Figure 12 and Figure 13, respectively. ADI’s HMC984 is chosen as the PFD component, whose input frequency reaches up to 150 MHz, and the output current is adjustable from 0.02 mA to 2.5 mA. ADI’s AD9915 is chosen as the DDS component, which has a maximum 64-bit frequency tuning word and a maximum system clock of 2.5 GHz. The experimental board runs the DDS with 2 GHz reference system clock and 32-bit frequency tuning word. In this situation, according to (24), the resolution of the DDS can be calculated in (28), corresponding to a 0.5 Hz frequency error.

(28) $$e_{precise}=\frac{F_{clk}}{2^{N}}=\frac{2\;\mathrm{GHz}}{2^{64}}\approx 0.5\;\mathrm{Hz}$$

In the specific experiment, the sampling rate of the system is set to 2 kHz, that is, the system completes a frequency measurement and outputs the result every 0.5 ms. Additionally, the tested signal generated by the signal generator is 80.000000 MHz. Considering the stability and timing requirements of system operation in FPGA, the reference signal $f_{ref}$ used in the equal-precision measurement module is set to 200 MHz. According to (25) and (26), the theoretical frequency measurement accuracy of the equal-precision method with a gate time of 0.5 ms is calculated as:

(29) $$\delta_{rough}=\frac{1}{T_{Gate}\cdot f_{ref}}=\frac{1}{0.5\times 10^{-3}\times 200\times 10^{6}}=1\times 10^{-5}$$

(30) $$e_{rough}=\delta_{rough}\cdot f_{x}=1\times 10^{-5}\times 80\times 10^{6}=8\times 10^{2}\;\mathrm{Hz}$$

**Figure 14:**
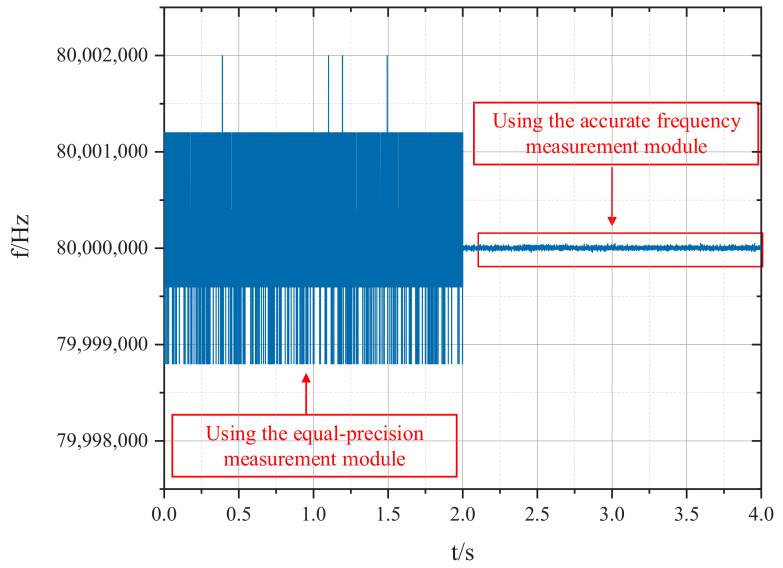
Comparison of frequency measurement results between the proposed method and the equal-precision method.

**Figure 15:**
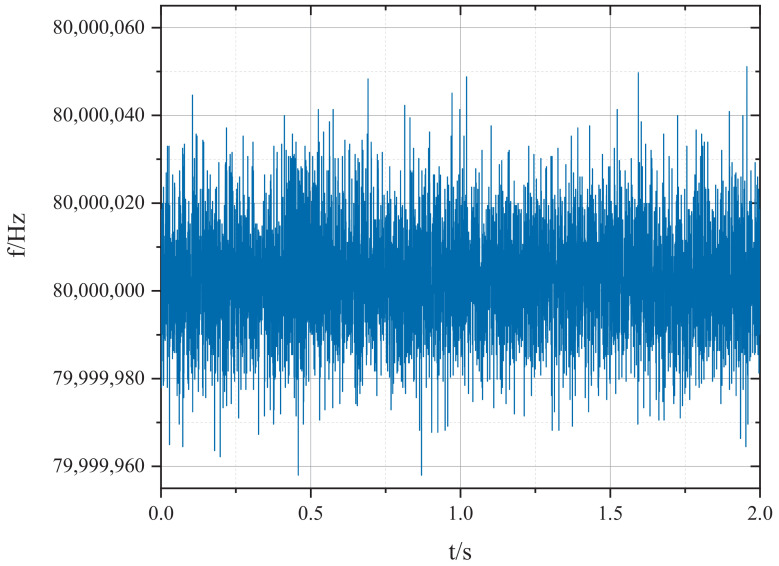
Detailed frequency measurement results of the proposed method.

In order to verify the effectiveness of the proposed system, the proposed frequency measurement method and the equal-precision frequency measurement method are used simultaneously to measure the 80.000000 MHz signal generated by the signal generator. The measurement results are compared in Figure 14. Obviously, the fluctuation of the measured frequency is greatly decreased after the introduction of the accurate frequency measurement module in the proposed system. The detailed frequency measurement results are shown in Figure 15. The comparisons of the measurement results are listed in Table 3. It can be seen from the table that the relative error of the frequency measurement system proposed in this paper is ±6.06000 × 10${}^{-7}$ under the sampling rate of 2 kHz, while the relative error of the equal-precision frequency measurement under the same sampling rate is only ±2.49813 × 10${}^{-5}$. Obviously, with the proposed method in this paper, the relative error and Allan variance are greatly optimized compared with the equal-precision frequency measurement method.

To sum up, it can be seen that the high-precision feedback frequency measurement system proposed in this paper can achieve a high-precision frequency measurement under the condition of high sampling rate. Additionally, in the case of a 2 kHz sampling rate, the relative error of the proposed method is reduced by more than 30 dB compared with the equal-precision measurement method, greatly improving the accuracy of frequency measurement.

## 5. Conclusions

With the development of different sensing mechanisms, a growing number of sensors are designed featuring high sensitivity to better detect signals. However, during the conversion of variations in physical quantities to the modulated frequency, high sensitivity has both pros and cons. On the one hand, high sensitivity can make the weak signal obtain greater gain. On the other hand, strong disturbance signals can be amplified as well, making the instantaneous frequency of the FM signal change drastically. Thus, the sampling rate must be increased to obtain the real-time variation information, which reduces the accuracy of frequency measurement accordingly and makes it hard to realize a high-precision measurement at a high sampling rate.

This paper presents a high-precision frequency measurement system based on negative feedback architecture. The system uses the PFD to realize the high-precision phase-to-frequency transformation and dynamically adjust the output of the DDS to track the tested signal. In this way, the quantization error is eliminated in principle, and the frequency measurement accuracy is independent of the sample time. The simulation results and the experiments have shown that the proposed system can effectively improve the accuracy of frequency measurement at a high sampling rate compared with the equal-precision measurement method. At a sampling rate of 2 kHz, the relative error of the proposed method is more than 30 dB better than that of the equal-precision measurement method. The proposed system can provide an effective solution for the high-precision frequency measurement when the frequency of the FM signal changes dynamically and there is a need for testing at a high sampling rate.

## Figures and Tables

**Table 1:** Simulation parameters of the key components in the model.

Frequency of the Tested Signal	Reference Signal ($f_{ref}$)	Sampling Gate Frequency	System Clock of DDS ($f_{clk}$)	Number of Bits in the Phase Accumulator (*N*)
10 kHz	200 kHz	1 Hz	64 kHz	26

**Table 2:** Simulated error table under the gate time of 1 s.

$f_{x}$/Hz	Frequency Measurement of the Proposed Method		Frequency Measurement by EQUAL-Precision Method	
	Max Absolute Error/Hz	Max Relative Error	Max Absolute Error/Hz	Max Relative Error
9999.93	0.001	$1\times 10^{-7}$	0.10	$1\times 10^{-5}$
11,000.03	0.001	$1\times 10^{-7}$	0.11	$1\times 10^{-5}$

**Table 3:** Frequency Measurement Accuracy and Stability of the System at 2 kHz Sampling Rate.

Method	Maximum	Minimum	Relative Error	Allan Variance @ 0.5 ms
Frequency measurement by equal precision method	80,001,998.81 Hz	79,998,799.72 Hz	±2.49813 × $10^{-5}$	3.77475 × $10^{-6}$
Frequency measurement of this system	80,000,048.79 Hz	79,999,957.99 Hz	±6.06000 × $10^{-7}$	8.75028 × $10^{-8}$

## Data Availability

The data that support the findings of this study are available from the corresponding author upon reasonable request.

## Abbreviations

The following abbreviations are used in this manuscript:

FM	Frequency modulated
DDS	Direct digital synthesizer
PFD	Phase–frequency detector
CP	Charge pump
LF	Loop filter
ADC	Analog-to-Digital converter
FPGA	Field programmable gate array
TIA	Trans-impedance amplifier
PID	Proportional–Integration–Derivative
